# Inhalant abuse: A clinic-based study

**DOI:** 10.4103/0019-5545.42399

**Published:** 2008

**Authors:** Suresh Kumar, Sandeep Grover, Parmanand Kulhara, Surendra Kumar Mattoo, Debasish Basu, Parthasarathy Biswas, Ruchita Shah

**Affiliations:** Department of Psychiatry, Drug De-addiction and Treatment Center, Postgraduate Institute of Medical Education and Research, Chandigarh - 160 012, India

**Keywords:** Inhalants, abuse, dependence

## Abstract

**Background:**

There are very few studies reporting inhalant abuse/dependence from India.

**Materials and Methods:**

Consecutive treatment seeking inhalant abuse cases (*n* = 21) were studied for the sociodemographic and clinical profile by using a semi-structured interview schedule.

**Results:**

A typical case profile was: unmarried male (100%), mean age 19 years, government school background (76%), unemployed (43%) or student (38%), urban nuclear family (86%), middle socioeconomic status (76%), and poor social support (62%); inhalant dependence (81%), inhalants being the only substance of abuse (33%) and of first or second preference (76%). Duration of inhalant use ranged 6-60 (mean 16) months. All subjects abused typewriter erasing fluid by sniffing (67%), huffing (19%) or bagging (14%). Initiation was out of curiosity (62%), under peer pressure (24%), or as a substitute (14%). Craving was more common (90%) than withdrawal (57%). Almost half of the cases (48%) had a family history for substance dependence. All cases were impaired, more so in family and educational/occupational domains.

**Conclusions:**

The results depict that easy availability, cheap price, faster onset of action, and a regular high makes inhalant a substance of abuse especially among the urban youth.

## INTRODUCTION

Inhalant abuse/dependence has been reported from various parts of the world.[1–4] Inhalants are volatile chemical vapors, which when inhaled produce a mind altering effect. They are found in substances like paint thinners, paint removers, dry cleaning fluids, glues, type writer correction fluids, gasoline, adhesives, varnishes, dry cleaning agents, deodorants, hair sprays, etc. When inhaled these chemicals are rapidly absorbed through the lungs into the blood and act on the brain and other organs, due to which, the user experiences intoxication. The signs and symptoms of intoxication may resemble those produced by alcohol. In addition, the user may experience light headedness, hallucinations, and delusions. Because of the short lasting effect, the abusers frequently seek the chemicals to prolong the high by repeated inhalations, which when done in excess can lead to loss of consciousness and death.[5] The literature from India is limited to only a few case reports[6–7] and case series.[8–11] This paucity of literature from India may be attributed to a lack of awareness among general population and health professionals regarding the abuse/dependence potential of inhalants and about it being a growing problem. In Indian literature, the commonly abused inhalants reported so far are petrol and typewriter print erasing fluid (TPEF). A typical inhalant abuser is a young adolescent either with scholastic decline[11] or as a school dropout,[9,12] comes from low to middle socioeconomic status family,[9,11,12] and abuses inhalants because of their easy accessibility, cheap price, faster onset of action, and the regular “high.”[11,12] To further understand the profile, the present research attempted to study the sociodemographic and clinical profile of the treatment seeking inhalant abusers.

## MATERIALS AND METHODS

The study was conducted at the Drug De-addiction and Treatment Center (DDTC) of the Department of Psychiatry at the Postgraduate Institute of Medical Education and Research, Chandigarh - a multispecialty institute in North India. At the DDTC, most patients come by self or family referral while some are referred from other hospitals or other departments of our Institute. The services provided by a team of psychiatrists, social workers, clinical psychologists, and nurses include outpatient, inpatient, laboratory, aftercare, and liaison with other governmental agencies and nongovernmental groups. The patients and their family members/attendants are assessed initially briefly by a psychiatrist and later in detail by a trainee psychiatrist who, after discussion with a Consultant Psychiatrist, finalizes the management plan and the diagnoses according to the ICD–10[13] and DSM-IV[14] . The planned management includes referral to/liaison with other departments, pharmacotherapy, psychotherapy, yoga-therapy, home visits, and socio-occupational rehabilitation. Regular follow-ups monitor and document the drug use profile, treatment issues, and the physical, psychological, social, and occupational functioning.

The study was approved by the departmental research review committee, which also gave ethical clearance. The study consisted of 21 DDTC cases registered and treated as either outpatients or inpatients between January 2002 and September 2005 for Inhalant abuse/dependence. The relevant case records were reviewed for the required data in accordance with the measures listed below.

### Measures

#### Socio-demographic data:

A semi-structured proforma was used to record sex, age, marital status, educational level, occupation, income, family type, religion, place of residence, and “family/social support system.” The last variable was included as suggested by another study from our center[14] and focused on key caregiver and significant figures in the family or in the society (peers, colleagues, job supervisors, etc.). “Poor support” was rated if support was unavailable. “Good/fair support” was rated when support was available from more than 1 member each from both the sources. The ratings were based on information recorded at the initial contact.

#### Clinical and substance use data:

This included the details of the substances used including order of preference, duration of use/abuse/dependence as per the DSM-IV,[14] relapses, treatments, and hospitalizations before the index treatment episode and physical and psychiatric comorbidity. The information about the comorbidity was inferred from the history, and physical and laboratory findings recorded all through the contact period. The substances were classified as primary, secondary, tertiary and fourth substance of use as per the self-report of patient in terms of preferential and predominant use.

#### Motivation:

The intent to give up inhalants or other substances was assessed on a 3-point grading: “0” for poor motivation, “1” for superficial/shallow, and “2” for fair/good motivation using a standardized guideline from a previous study from our center.[15]

#### Impairment data:

Four levels of substance related impairment recorded at the first presentation were operationalized for the areas of health, education/occupation, finance, family, marital, legal, and social life. The severity of complications (nil, mild, moderate, and severe) was extracted from the case records using a standardized guideline from a previous study from our center.[15]

#### Time in treatment (Duration of follow-up):

This was calculated as number of months between the first and the last visit to the hospital; including the duration of inpatient stay, if any.

#### Outcome:

Abstinence, lapse, or relapse were considered the primary outcome measures as recorded at the last follow-up. Abstinence was defined as no substance intake. Lapse was defined as use of the substance less than that for relapse. Relapse was defined as re-emergence of substance dependence as per the DSM-IV.[14]

## RESULTS

### Inhalant abuse/dependence: Increase over the years

Between 2002 and 2005, a steady increase in the cases was recorded: from one case in 2002 (out of 710 cases registered; 0.0014%), four cases in 2003 (out of 886 cases registered; 0.0045%), nine cases in 2004 (out of 954 cases registered; 0.0094%), to seven cases between January and September 2005 (out of 767 cases registered; 0.0091%). This reflects increase in both the absolute numbers as also the percentage out of the total DDTC case load. However, the data could not be analyzed to see the statistical significance in terms of trends because of only one case in one of the years under study. Five out of the 21 cases included in this study were earlier reported in a case series.[11]

All 21 cases were unmarried men aged 12-27 years, out of which three (14%) were < 14 years at presentation, eight (38%) were between 15 and 18 years of age at first presentation. While 42.9% (*n* = 9) were unemployed and 38% (*n* = 8) were students, only 19% (*n* = 4) were employed. Out of the students, 75% (six out of eight cases) were irregular in attending schools. All other cases, whether employed or unemployed, were school drop-outs. About three-quarters (76.2%) of the cases had attended government run schools. The socioeconomic status was low in 23.8% cases and middle in 76.2% cases. A typical case presented at the mean age of 19.16 ± 4.05 years, came from urban nuclear family (85.7% each), and had poor social support (61.9%). Five subjects (24%) came from a single parent family. While 76.2% cases were brought to the center by a relative, 23.8% were referred by doctors.

### Clinical profile

At initial presentation 17 (81%) cases met dependence criteria of DSM-IV and four subjects (19%) fulfilled the criteria of abuse as per DSM-IV. In seven (33.33%) cases, inhalant was the only substance of dependence/abuse. Other substances of abuse included nicotine, cannabis, carisoprodol, alcohol, and opioids (heroin, dextropropoxyphene, and buprenorphine).

As a substance of abuse, the first and second preference was for inhalant in 11 and 5 cases, for cannabis in 5 cases each and for nicotine in 3 and 5 cases, respectively. Similarly, third preference was for inhalant and nicotine in four cases each and alcohol and buprenorphine in one case each, and fourth preference was for inhalant, buprenorphine and cannabis in one case each. Inhalant was the first-in-life drug in eight cases (38%); out of these, two used nicotine and one used cannabis later on. Out of 21, only six subjects were treated as inpatients.

Compared to the mean age at first presentation of 19.16 ± 4.05 years (range: 12-27 years), the mean age at first use of the inhalant was 17.35 ± 4.06 years (range: 11.5-27.0 years). Thus, the mean duration of inhalant use at presentation was 16.31 ± 16.81 months (range: 0.5–60.0 months).

All 21 cases were using TPEF; one case also sniffed petrol vapors. TPEF used ranged between 5 and 75 ml (Mean ± SD: 40.29 ± 18.83 ml) per day. The modes of use were: sniffing from a container (66.7%), huffing from a cloth (19.0%), and bagging (breathing fumes from a plastic bag held tightly around the mouth, 14.3%). Initiation of inhalant use was out of curiosity (61.9%), under peer pressure (23.8%) or as a substitute for another substance (14.3%). Two-third (66.66%) subjects reported an immediate intoxication as “kick/high/euphoria/feeling of relaxation” with one or more of the following symptoms: giddiness (23.8%), unsteadiness or perceptual disturbances (19.0% each), unconsciousness or delirium (14.3% each) and lightheadedness (9.5%). Craving was reported by 90.5% cases. One or more of the following withdrawal symptoms were reported by 57.1% cases only: irritability (23.8%), subjective restlessness (14.3%), observed restlessness (9.5%), and insomnia, tingling sensation all over the body, headache and poor concentration (4.8% each). Comorbid seizure disorder was recorded in 9.5% cases. The recorded comorbid psychiatric disorders included conduct disorder (19%) and schizophrenia (4.8%). Two subjects reported developing psychosis while using the inhalant as primary substance of use, including one case who was also using cannabis. Out of the 21 cases, five had comorbid psychiatric illness, most common being a diagnosis of conduct disorder (*n* = 4). A positive family history was recorded for substance dependence in nearly half of the cases (47.6%), which included nicotine (23.8%), alcohol (19%), and opioids (4.8%). Motivation for treatment was recorded as “good” in 19% and “superficial” or “poor” in 81% cases. Out of the six cases treated as inpatients, four had additional diagnosis of other substance dependence including benzodiazepine, cannabis, opioids, and cannabis plus opioid (4.8% each).

### Impairment

All cases reported some impairment in family and educational/occupational domains. No impairment was recorded in legal (81% cases), social (23.8% cases), financial (19% cases), and physical (14.3% cases) domains. Impairment was mostly mild-to-moderate in all domains except the legal domain. Severe impairment was reported only in the domains of finance (28.6% cases), education/occupation (19% cases), and physical (14.3% cases).

### Outcome

The mean duration of follow-up was 1.84 ± 3.26 months (range: 0-14 months). The mean number of follow-up hospital visits was 3.47 ± 4.68 (range: 1-20). At the last follow-up, 42.9% cases were reportedly abstinent, while 57.1% cases were continuing to take inhalant or had relapsed.

## DISCUSSION

As per our observation, 21 cases sought treatment from our center; the number of inhalant-abusers with us has increased over the years; most of these knew others in the community who were into inhalant abuse implying that there are many more such cases in the community; there is a gradual increase in academic research[6–11] and the lay media output on this issue.[16]

The reasons for the increase in inhalant abuse/dependence are: easy availability (at all stationary/ general stores, with no legal control over the sale), low cost, small/ easy to hide container, quick and definite “high,” and lack of awareness of the abuse/dependence potential on the part of the public-sellers and parents in particular (TPEF is accepted as something useful for studies and office work).

All our cases being male does not necessarily mean that females are not involved; lack of awareness and/or greater stigma attached to substance abuse among the females may explain their absence.

Most of our cases coming from urban locality may be a reflection of increase in urbanization, accessibility to treatment or true prevalence of inhalant abuse/dependence in urban population.

Majority of our subjects being either school dropouts or irregular to the school is a finding similar to earlier reports.[9,12] It may reflect the possibility of impaired cognitive functions, lower scholastic performance and school drop out, especially if the substance abuse starts in preadolescence.

Our findings of positive family history of substance abuse, and poor social support/supervision conforms to the western literature on inhalant abusing children.[17] Most of our cases coming from middle socioeconomic status may reflect the treatment-seeking pattern/ability rather than the true community prevalence of the inhalant abuse.

The commonest reasons for first use in our cases being curiosity or peer pressure is similar to other reports from India.[12] Majority of our subjects abusing other substances too, most commonly nicotine and cannabis, probably reflects the true drug use pattern in the community. This conclusion is supported by previous research from our area[9] and other parts of India.[12]

Previous research has reported inhalant abuse as a predictor of future polysubstance abuse, particularly of intravenous drugs.[4,18,19] In 38% of our subjects for whom inhalant was the first-in-life drug, 10% progressed to nicotine abuse and 5% to cannabis abuse; ≤ 2 month follow-up in our cases leaves the possibility of future polysubstance abuse open.

Craving reported by most of our cases indicates that inhalants carry a potential for abuse or dependence. The intoxication and withdrawal profiles reported by our subjects are similar to the ones reported earlier.[5] The development of psychosis in two of our subjects seems to reflect the hypothesized brain dopaminergic activation, and glutamatergic and GABA-ergic inhibition.[5]

Excessive generalization of our findings is unwarranted for many reasons; we focused only on treatment seekers; carried out retrospective chart review; some of our instruments and definitions (e.g., social support, impairment, motivation, and outcome) are study/center specific and untested for reliability; and, findings are attributable to other substances being used or abused as well.

The study of the profile of the patients attending a de-addiction clinic is useful in a number of ways. It can guide the organization of treatment services and preventive steps including the legal control of production, distribution, and sale and purchase of such substances. The contribution assumes greater importance when the representative national level epidemiologic data is missing in India and most of the developing countries. There is also a need to debate the necessity to enact legislation to limit access to inhalants.
